# Monitoring blood potassium concentration in hemodialysis patients by quantifying T-wave morphology dynamics

**DOI:** 10.1038/s41598-021-82935-5

**Published:** 2021-02-16

**Authors:** Flavio Palmieri, Pedro Gomis, Dina Ferreira, José Esteban Ruiz, Beatriz Bergasa, Alba Martín-Yebra, Hassaan A. Bukhari, Esther Pueyo, Juan Pablo Martínez, Julia Ramírez, Pablo Laguna

**Affiliations:** 1 Centre de Recerca en Enginyeria Biomèdica, Universitat Politècnica de Catalunya, Barcelona, Spain; 2 CIBER en Bioingeniería, Biomateriales y Nanomedicina (CIBER-BBN), Zaragoza, Spain; 3 Laboratorios Rubió, Castellbisbal, Barcelona Spain; 4 Valencian International University, Valencia, Spain; 5 Nephrology Department, Hospital Clínico Universitario Lozano Blesa, Zaragoza, Spain; 6 BSICoS Group, I3A, IIS Aragón, Universidad de Zaragoza, Zaragoza, Spain; 7 William Harvey Research Institute, Queen Mary University of London, London, UK

**Keywords:** Biomarkers, Cardiology, Diseases, Risk factors, Engineering, Mathematics and computing

## Abstract

We investigated the ability of time-warping-based ECG-derived markers of T-wave morphology changes in time ($d_{w}$) and amplitude ($d_a$), as well as their non-linear components (${d_w^{{\mathrm{NL}}}}$ and ${d_a^{\mathrm{NL}}}$), and the heart rate corrected counterpart ($d_{w,c}$), to monitor potassium concentration ($[K^{+}]$) changes ($\Delta [K^+]$) in end-stage renal disease (ESRD) patients undergoing hemodialysis (HD). We compared the performance of the proposed time-warping markers, together with other previously proposed $[K^{+}]$ markers, such as T-wave width ($T_w$) and T-wave slope-to-amplitude ratio ($T_{S/A}$), when computed from standard ECG leads as well as from principal component analysis (PCA)-based leads. 48-hour ECG recordings and a set of hourly-collected blood samples from 29 ESRD-HD patients were acquired. Values of $d_w$, $d_a$, ${d_w^{\mathrm{NL}}}$, ${d_a^{\mathrm{NL}}}$ and $d_{w,c}$ were calculated by comparing the morphology of the mean warped T-waves (MWTWs) derived at each hour along the HD with that from a reference MWTW, measured at the end of the HD. From the same MWTWs $T_w$ and $T_{S/A}$ were also extracted. Similarly, $\Delta [K^+]$ was calculated as the difference between the $[K^{+}]$ values at each hour and the $[K^{+}]$ reference level at the end of the HD session. We found that $d_{w}$ and $d_{w,c}$ showed higher correlation coefficients with $\Delta [K^+]$ than $T_{S/A}$—Spearman’s ($\rho$) and Pearson’s (*r*)—and $T_w$—Spearman’s ($\rho$)—in both SL and PCA approaches being the intra-patient median $\rho \ge 0.82$ and $r \ge 0.87$ in SL and $\rho \ge 0.82$ and $r \ge 0.89$ in PCA respectively. Our findings would point at $d_{w}$ and $d_{w,c}$ as the most suitable surrogate of $\Delta [K^+]$, suggesting that they could be potentially useful for non-invasive monitoring of ESRD-HD patients in hospital, as well as in ambulatory settings. Therefore, the tracking of T-wave morphology variations by means of time-warping analysis could improve continuous and remote $[K^{+}]$ monitoring of ESRD-HD patients and flagging risk of $[K^{+}]$-related cardiovascular events.

## Introduction

Chronic kidney disease (CKD) is defined as the presence of kidney damage, persisting for 3 months or more, irrespective of the cause^1^. It represents a state of progressive loss of kidney function ultimately resulting in need for renal replacement therapy such as hemodialysis (HD) or transplantation. The development of CKD and its progression to this terminal stage, called end-stage renal disease (ESRD), remains a significant source of reduced quality of life and premature mortality^2^. In particular, sudden cardiac death (SCD) represents an important cause of death in ESRD-HD patients^3^. Various risk factors may be responsible for SCD in this patient population, including left ventricular hypertrophy and fibrosis, disordered bone-mineral metabolism, HD-induced changes in electrolyte, and fluid and acid-base status, which may lead to electrocardiographic (ECG) abnormalities and ventricular arrhythmia^3,4^.

Recent studies have shown that blood potassium concentrations ($[K^{+}]$) outside the physiological interval are associated with increased mortality risk^5^. In healthy conditions, the maintenance of $[K^{+}]$ homeostasis is ensured by normal renal activity^6^. However, ESRD-HD patients suffer from $[K^{+}]$ imbalance, leading to a high incidence of arrhythmic events. The pro-arrhythmic consequences of $[K^{+}]$ imbalance can be explained considering that potassium currents are involved in the repolarization process of the cardiac action potential (AP), determining membrane potential and refractoriness of the myocardium^7^. Therefore, even modest deviations of $[K^{+}]$ from its normal range (hypokalemia if $[K^{+}]$ < 3.5 mmol/L or hyperkalemia if $[K^{+}]$ > 5 mmol/L) may lead to hospitalisation or death in ESRD-HD patients^8^. Evaluation of $[K^{+}]$ levels is currently based on blood samples that require further analyses in the laboratory, limiting continuous monitoring. Non-invasive markers able to track variations in $[K^{+}]$ levels are therefore needed.

The electrocardiogram (ECG) is a non-invasive, easily accessible, and inexpensive practice that reflects the electrical activity of the heart. In particular, the T-wave reflects the spatio-temporal repolarization of the ventricle, and its analysis has been used to measure the vulnerability of a patient to ventricular arrhythmias^9^. This fact is of particular interest because T waves are frequently altered in ESRD-HD patients^4^. The QT interval is the standard index of ventricular repolarization, and it has been proposed to monitor ESRD-HD patients^10^. However, the effects of HD on QT interval, and its corrected version QTc, are still controversial, since several studies^11^ reported a prolongation during the HD sessions, but others reported opposite trend or even no changes at all^12^. This motivates the analysis of the overall T-wave morphology as a potential potassium level marker.

Different T-wave morphology markers have been previously reported to be correlated with $[K^{+}]$, such as the T-wave right slope^13^, the width of the T-wave ($T_{w}$)^14^, the T-wave slope-to-amplitude ratio ($T_{S/A}$)^15^, and a morphology combination score, which integrates features like T-wave asymmetry, flatness and notching^16^. However, these markers rely on specific local features of the T-wave rather than in the overall T-wave morphology, which may have a stronger potential in following $\Delta [K^+]$ than indices based on local features.

A recent study reported a time-warping based methodology to quantify changes in the overall T-wave morphology^17^. Six indices were proposed, $d_{w}^{u}$ and $d_a$, reflecting morphological variations in time and amplitude, respectively, as well as their non-linear version, $d_w^{\mathrm{NL}}$ and $d_a^{\mathrm{NL}}$ as reported in^17^ and two novel markers derived from $d_{w}^{u}$ and named $d_{w}$ and ${d}_{w,c}$. The main goal of this study is to investigate the potential of these markers in monitoring both hypo- and hyperkalemia events excluding the variability due to the heart rate (HR) and to compare their performance against $T_{w}$ and $T_{S/A}$ in standard single-lead approach and by applying principal component analysis (PCA) as multilead space reduction technique. However, some of the above mentioned indices may not be robust enough for our purpose. It is the case of $d_w$ which does not provide information about the direction of the T-wave morphological variation (i.e. if there is stretching or shortening) and has been found to be correlated with HR. Therefore, we have adapted the original methodology^17^ to account for hypo- and hyperkalemia, and we propose a new marker that is independent of HR, thus offering a more precise $[K^{+}]$ monitoring tool for arrhythmic risk stratification in ESRD-HD patients. Preliminary results extracted from a smaller subset of patients have been presented at Computing in Cardiology conference^18,19^ while the electrophysiological basis was studied in Bukhari et al.^20^.

The novelties of the present study with respect to the state-of-the-art are: (1) the usage of T-wave time warping analysis for non-invasive $[K^{+}]$ monitoring, together with the development of a HR correction tool for the time-warping marker, ${d}_{w,c}$; (2) the proposal of a PCA spatial transformation lead for marker extraction and (3) the validation of the proposed markers in comparison with previously published biomarkers ($T_{w}$ and $T_{S/A}$) and with their extraction from standard leads.

## Materials and methods

### Study population

The study population included 29 patients from the Nephrology ward from Hospital Clínico Universitario Lozano Blesa (Zaragoza, Spain). Inclusion criteria were (i) 18-year-old (or older), (ii) having a diagnosed ESRD pathology and (iii) undergoing HD at least three times per week (with venous or cannula access). Table 1 shows the population characteristics. The study protocol was approved by the Aragon’s research ethics committee (*CEICA*, ref. PI18/003) and all patients and/or their legal guardians signed informed consent. All the procedures and all the methods were performed in accordance with the Helsinki Declaration. The database collection is still ongoing, with the current size significant enough for a pilot study^21,22^.

**Figure 1:**
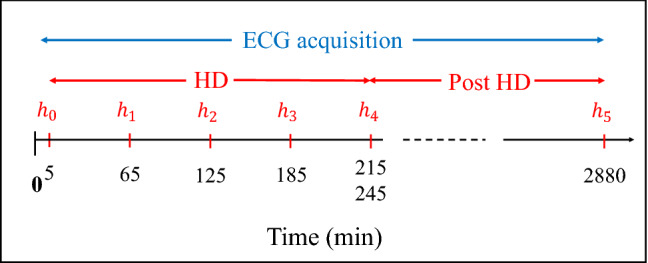
Diagram of the study protocol: $h_0$ to $h_5$ are the time points (in minutes) for blood sample extraction. $h_4$ is taken at the end of the HD (minute 215-th or 245-th, depending on the HD duration).

**Figure 2:**
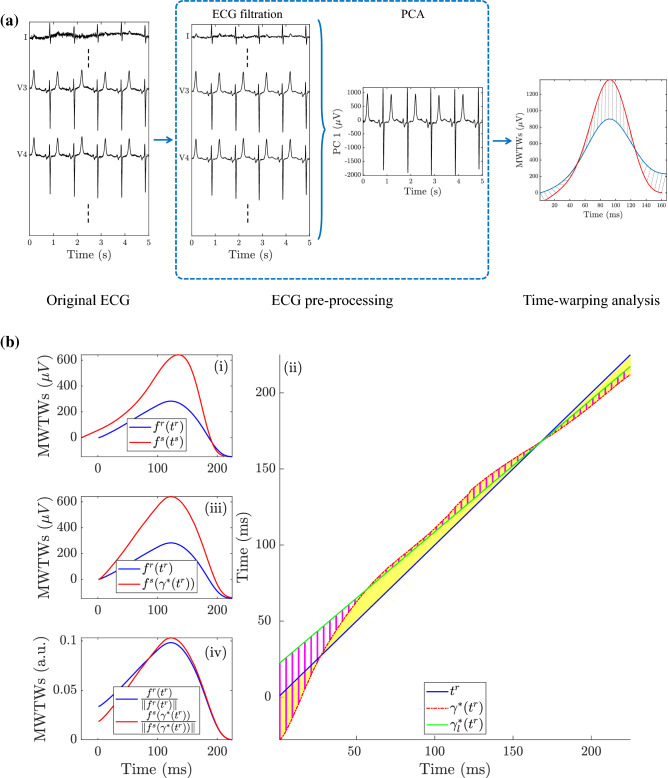
Analysis stages performed in this study. In panel (**a**) is the flow chart showing the ECG processing steps for T-wave time-warping markers extraction. The analysis starts with the original ECG, followed by a filtering step before spatial PCA analysis, to conclude with markers computation. Panel (**b**) shows an example of the linear and nonlinear time-warping markers for the same patient as in Fig. 5a. In particular, subpanel (i) shows both the reference (blue) and the *i*-th MWTW (red) while subpanel (ii) shows the warping function (red dotted line) that optimally relates the reference and studied MWTWs. Subpanel (iii) shows the MWTWs after warping and subpanel (iv) are the normalized reference and warped MWTWs.

### Data collection

#### General information

Sex, age, concomitant therapies (e.g. assumption of anti-arrhythmic drugs), kidney disease etiology and HD treatment related information were collected for each enrolled patient, as detailed in Table 1.

#### Blood sample analysis

For each patient, six blood samples were taken and analysed during the HD session: the first one at the HD onset and the next three, every subsequent hour (Fig. 1, $h_0$ to $h_3$ in red). The 5-th blood sample was collected at the end of the HD (minute 215-th or 245-th, depending on the HD session duration) while the 6-th blood sample was taken after 48 h, immediately before the next HD session. Potassium, magnesium, calcium, urea, creatinine, bicarbonate and pH were measured from each blood test. Blood potassium concentrations values for each blood test are given in Table 2.

#### ECG measurements

A 48h, standard 12-lead ECG Holter recording, (H12+, Mortara Instruments, Milwaukee, WI, USA, sampling frequency of 1 kHz, amplitude resolution of 3.75 $\upmu$V), was obtained for each enrolled patient, starting the acquisition 5 min before the HD onset (Fig. 1, blue line). The block diagram presented in Fig. 2a describes the main steps of the whole data processing implemented in this work.

### ECG pre-processing

#### ECG filtering

Holter ECG signals contain baseline drift and other noises, such as power-line and muscular activity (Fig. 2a). Therefore, an initial pre-processing is needed to improve the signal-to-noise ratio (SNR) and enable ECG waveform analysis. First, baseline wander was removed with a high-pass, forward-backward 6-th order Butterworth filter with 0.5 Hz cut-off frequency^23^ (Fig. 2a). Then, residual noise out of the T-wave band was removed with a 6-th order low-pass Butterworth filter with 40 Hz cut-off frequency.

#### ECG waveform detection and delineation

A wavelet-based single-lead method^24^ was applied to detect QRS complexes and then delineate T-wave onsets and ends in each of the 12 leads. The wavelet transform (WT) decomposes the signal in the time-scale domain, allowing its representation at different resolutions. It is, therefore, a suitable tool to analyze ECG signals, which contain patterns with different frequency content (QRS complexes, P and T-waves).

#### Single-lead delineation

The discrete dyadic WT is implemented in such a way that it keeps temporal resolution at different scales. The detection of the fiducial points is carried out across the adequate WT scales, attending to the dominant frequency components of each ECG wave: Q,R,S waves correspond to a simultaneous effect in scales $2^1$–$2^2$, while the T and P waves affect mainly scales $2^4$ or $2^5$, see^24^ for details. ECG wave peaks correspond to zero crossings in the WT, and ECG maximum slopes correspond to WT’s maxima and minima. Depending on the number and polarity of the slopes found, a wave morphology is assigned and boundaries are located using threshold-based criteria. The onset (end) of a wave occurs before (after) the first (last) significant slope associated with the wave^24^.

#### Selection rules for multi-lead delineation

To obtain multilead peak locations, a median post-processing selection rule over the single-lead-based detected locations is used. The post-processing rules for boundaries consist of ordering the single-lead annotations and selecting as the onset (end) of a wave the first (last) annotation whose *k* nearest neighbours lay within a $\delta$ ms interval^24,25^.

#### Single-lead analysis

First, we performed the analysis using the single-lead ECG, taking the T-waves from leads V3 to V6, as used in a previous study^26^ for $[K^+]$ estimation, and lead II being the most widely used in patient monitoring^27^. These T waves were further delineated by using the above motioned delineator^24^ and the biomarker estimation is performed as described below in section named “Time warping analysis”.

#### Spatial lead reduction by principal component analysis

Next, a spatial lead reduction by Principal Component Analysis (PCA) was made since it was found to be a robust spatial transformation to emphasize waveform SNR^28^. In this work PCA was spatially applied to the 8 independent leads, learned over the T-wave segment to mainly emphasize this waveform, and resulting in 8 principal components (PCs) or transformed leads. The coefficients defining the PCA transformation were obtained from the eigenvectors of the $8 \times 8$ interlead auto-correlation matrix computed over the T-waves in a 10-min wide window at the end of the HD session. The correct delineation of T-waves is crucial to emphasize only T-wave energy content. The first PCA, denoted as PC1, was used for the subsequent ECG analysis, as it is the transformed lead where the T-waves have maximal energy, and thus, maximal SNR for morphological characterisation^28,29^. PC1 was further delineated by applying again^24^, and each T-wave was further low-pass filtered at 20 Hz using a 12-th order Butterworth filter to restrict shape analysis to the dominant band of the T-wave so removing remaining noisy components that could still corrupt the T-wave shape analysis.

### Time-warping analysis

Two-minute ECG segments, centred on the 5-th and 35-th minutes of each available hour, were analysed. The window duration was short enough to hold the assumption of stability for both $[K^+]$ and HR values. Figure 5a shows the average RR interval for each selected *i*-th 2-min segments for a given patient along the ECG recording. While the blood samples (purple diamonds) were collected each hour during the HD, the warping parameters were computed every half an hour to get a more detailed view over time.

For each *i*-th 2-min segment, a mean warped T-wave (MWTW) was computed. First of all, the predominant T-wave polarity (e.g. upward, downward etc) within a given window, was defined as that having the highest number of occurrences. This polarity change can be physiological or induced by delineator oscillation when by-phasic to regular T-waves appears almost indistinguishable. A T-wave was considered to have inverted polarity if the magnitude of its peak had negative sign and vice-versa. Only those T-waves having the same polarity as the predominant one were considered in the following steps. Then, all these selected T-waves were aligned with respect to their gravity center and used to compute an initial MWTW^17^. Finally, all the T-waves were checked to find and discard the outliers, defined as having T-wave duration outside the range $T_{dm_i} \pm 1.5\times \sigma _{d_i}$ centered at the *i*-th ensemble T-wave mean, $T_{dm_i}$, and bounded function of the T-wave duration standard deviation $\sigma _{d_i}$. Among the remaining, only those T-waves highly correlated (Pearson’s correlation coefficient > 0.98) with the previous initial MWTW were used to recalculate the final MWTW. The MWTW at the end of the HD treatment was taken as the reference, given that it is the time when the patient (a) is supposed to have recovered the normal $[K^+]$ level and (b) was discharged from hospital, being an appropriate reference for out-of-hospital ambulatory monitoring.

Since hyperkalemia has been reported to cause T-wave inversions^30^, any MWTWs with negative-polarity was inverted before performing the warping with the reference MWTW. Previous to warping, the two MWTWs were aligned with respect to their gravity center, so that only changes in the T-wave morphology, and not those associated with their relative delay, were quantified by the warping algorithm.

For comparison purposes, both $T_w$^14^ and $T_{S/A}$^15^ were extracted from each MWTW and their performance, with respect to T-wave time-warping based biomarkers in monitoring $[K^+]$, was assessed. This work perform a clinical study following previous analysis testing the marker by electrophysiological simulations as reported in Bukhari et al.^20^.

#### T-wave time warping

The method here applied was originally proposed by Ramírez et al.^17^. Let ${{\mathbf {f}}^i(\mathbf {t}^i)=[f^i(t^i(1)),\ldots ,f^i(t^i(N_i))]^T}$ be the MWTW of a given *i*-th segment, and ${\mathbf {f}^r(\mathbf {t}^r)=[f^r(t^r(1),\ldots ,f^r(t^r(N_r))]^T}$ the reference MWTW, where ${{\mathbf {t}}^i =[t^i(1),\ldots ,t^i(N_i)]^T}$ and ${{\mathbf {t}}^r =[t^r(1),\ldots ,t^r(N_r)]^T}$ with $N_i$ and $N_r$ being the total T-wave duration, in samples, of ${{\mathbf {t}}}^i$ and ${\mathbf {t}}^r$ respectively. Figure 2b illustrates the warping method applied between one of the *i*-th MWTW (red) and the reference MWTW (blue). Let $\gamma _{i}({\mathbf {t}}^r)$ be the warping function that relates ${\mathbf {t}}^r$ and ${\mathbf {t}}^i$, such that the composition $({\mathbf {f}}^i\circ \gamma _{i})({\mathbf {t}}^r)$ denotes the re-parametrization or time domain warping of $\mathbf {f}^i({\mathbf {t}}^i)$ using $\gamma _{i}({\mathbf {t}}^r)$, i.e. $(\mathbf {f}^i\circ \gamma _i)({\mathbf {t}}^r)$ represents the amplitude values of ${\mathbf {f}}^i({\mathbf {t}}^i)$ if its temporal vector was ${\mathbf {t}}^r$. The square-root slope function (SRSF) was proposed instead of the original T-waves^31,32^ to find the optimal warping function. This was applied by performing time-warping on the SRSFs of the T-waves, preventing the “pinching effect” in cases when T-wave amplitudes differ^33^. This transformation is defined as:

1 $$\begin{aligned} \mathbf {q}_{f}\left( {\mathbf {t}} \right) =\hbox {sign}\left( \dot{\mathbf {f} }\left( {\mathbf {t}} \right) \right) \sqrt{\left| \dot{{\mathbf {f}} }\left( {\mathbf {t}} \right) \right| }. \end{aligned}$$

The optimal warping function is the one that minimizes the amplitude difference between the SRSF of ${\mathbf {f}}^r({\mathbf {t}}^r)$ and ${\mathbf {f}}^i(\gamma _i({\mathbf {t}}^r))$^32^:

2 $$\begin{aligned} \gamma _i^*\left( \mathbf {t}^r\right)&=\underset{\gamma _{i}\left( {\mathbf {t}}^r\right) }{\arg \min } \left( \left\| {\mathbf {q}}_{f^r}\left( {\mathbf {t}}^r\right) -\mathbf{q}_{[f^i\circ \gamma _i]}\left( {\mathbf {t}}^r\right) \right\| \right) \nonumber \\&=\underset{\gamma _i\left( \mathbf {t}^r\right) }{\arg \min }\left( \left\| {\mathbf {q}}_{f^r}\left( \mathbf {t}^r\right) -{\mathbf {q}}_{f^i}\left( \gamma _i\left( \mathbf {t}^r\right) \right) \sqrt{\dot{\gamma _i}\left( \mathbf {t}^r\right) }\right\| \right). \end{aligned}$$

The dynamic programming algorithm was used to obtain the solution of this optimisation problem^34^. Figure 2b(ii) shows the optimal warping function between the two waves in Fig. 2b(i). The warped T-wave, ${\mathbf {f}}^i(\gamma ^*_{i}({\mathbf {t}}^r))$ is shown in Fig. 2b(iii), together with the reference T-wave, ${\mathbf {f}}^r({\mathbf {t}}^r)$.

#### Time warping biomarkers

The index $d_{w}^{u}$ (corresponding to the index denoted as $d_{w}$ in^17^), shown as the yellow area in Fig. 2b(ii), quantifies the amount of warping needed to optimally align the two T-waves, and is defined as the average of the absolute difference value between $\gamma _i^*({\mathbf {t}}^r)$ and ${\mathbf {t}}^r$:

3 $$\begin{aligned} d_{w}^{u} (i)=\frac{1}{N_r}\sum _{n=1}^{N_r} |\gamma ^*_{i}(t^r(n))-t^r(n)|. \end{aligned}$$

The original definition of $d_{w}^{u}(i)$^17^ was modified here to allow the marker to be signed, therefore distinguishing T-wave widenings from narrowings. This signed $d_{w}(i)$ was defined as:

4 $$\begin{aligned} d_{w}(i)=\left( \frac{s_d(i)}{|s_d(i)|}\right) \frac{1}{N_r}\sum _{n=1}^{N_r} |\gamma ^*_{i}\left( t^r\left( n\right) \right) -t^r\left( n\right) |. \end{aligned}$$

where $s_d(i)$ was used to account for the sign of the $d_{w}(i)$ and it was computed as:

5 $$\begin{aligned} s_d(i)= \sum _{n \in N_r^u} (\gamma ^*_{i}\left( t^r\left( n\right) \right) -t^r\left( n\right) ) + \sum _{n \notin N_r^u} (t^r\left( n\right) -\gamma ^*_{i}\left( t^r\left( n\right) \right) ). \end{aligned}$$

with $N_{r}^{u}$ being the set of T-wave up-slope samples. A positive sign means that the ${\mathbf {f}}^i({\mathbf {t}}^i)$ has to be widened to fit the ${\mathbf {f}}^r({\mathbf {t}}^r)$ and vice-versa for a negative sign.

After applying time warping between both MWTWs, the amplitude difference between ${\mathbf {f}}^r({\mathbf {t}}^r)$ and $\mathbf {f}^i(\gamma ^*_{i}({\mathbf {t}}^r))$ is quantified as the area contained between ${\mathbf {f}}^r({\mathbf {t}}^r)$ and $\mathbf {f}^i(\gamma ^*_{i}({\mathbf {t}}^r))$, normalized by the L2-norm of ${\mathbf {f}}^r({\mathbf {t}}^r)$:

6 $$\begin{aligned} d_{a}(i) =\frac{s_a(i)}{\Vert s_a(i) \Vert } \frac{\Vert \mathbf {f}^i(\gamma _i^*({\mathbf {t}}^r))- {\mathbf {f}}^r({\mathbf {t}}^r)\Vert }{\Vert \mathbf {f}^r({\mathbf {t}}^r)\Vert } \times 100. \end{aligned}$$

where $s_a (i)=\sum _{n=1}^{N_{r}} ( f^i(\gamma _i^*(t^r))- f^r(t^r))$ is used to account for the $d_{a}(i)$ sign estimation.

Both $d_{w}(i)$ and $d_{a}(i)$ incorporate information from the linear and non-linear differences between both T-waves in time and amplitude domain, respectively. The non-linear components can be quantified as in^17^:

7 $$\begin{aligned} d_{w}^{\mathrm{NL}}(i)= & {} \frac{1}{N_r}\sum _{n=1}^{N_r} |\gamma _i^*(t^r(n))- \gamma ^*_{i,l}(t^r(n))|. \end{aligned}$$

8 $$\begin{aligned} d_{a}^{\mathrm{NL}}(i)= & {} \biggl \Vert \frac{\mathbf {f}^r({\mathbf {t}}^r)}{\Vert {\mathbf {f}}^r({\mathbf {t}}^r)\Vert } - \frac{\mathbf {f}^i(\gamma _i^*({\mathbf {t}}^r))}{\Vert {\mathbf {f}}^i(\gamma _i^*(\mathbf {t}^r))\Vert } \biggr \Vert \times 100. \end{aligned}$$

where $\gamma ^*_{i,l}({\mathbf {t}}^r)$ (green line in Fig. 2b(ii)) is the best linear fitting to $\gamma _i^*({\mathbf {t}}^r)$ according to the least absolute residual criterion^35^. The parameter $d_w^{\mathrm{NL}}(i)$ quantifies the non-linear warping by computing the area of the dashed magenta region between $\gamma ^{*}\left( {\mathbf {t}}^r\right)$ and $\gamma ^{*}_{i,l}\left( {\mathbf {t}}^r\right)$ (in Fig. 2b(ii)). Finally, the marker $d_{a}^{\mathrm{NL}} (i)$ quantifies the residual information in amplitude domain after normalising MWTWs (Fig. 2b(iv)).

#### Heart-rate-corrected T-wave warping

It is well known that T-wave duration and QT interval are strongly dependent on HR^36^. Although aligning the T-waves according to their gravity centre reduces most of the dependence of $d_w(i)$ on HR, there may still be some residual dependence in T-wave morphology that should be compensated for (e.g. see Fig. 5a around hours h = 9, 12 and 43). We assume that $d_w(i)$, as originally proposed in (4), can be modelled as the sum of two components:

9 $$\begin{aligned} d_{w}(i)= d_{w,c}(i)+d_{w,HR}(i). \end{aligned}$$

where $d_{w,HR}(i)$ is the HR dependent component and $d_{w,c}(i)$ is the non-HR dependent component accounting for ($K^{+}$) induced variations and possibly others not HR related.

To estimate the corrected component $d_{w,c}(i)$ we depart from the literature, where several formulae for HR-dependency correction of repolarization related time intervals, like the QT interval, have been developed^37–40^, including a variety of approaches (e.g. linear, hyperbolic, exponential models etc.) being investigated and tested in view of the complex relationship between QT interval and HR^39^. To derive a correction formula and estimate $d_{w,c}(i)$, we started from a linear approximation of a hyperbolic model under small RR changes, derived similarly to the QT interval correction (QTc)^38,39^,

10 $$\begin{aligned} QT= \beta (RR)^{\alpha }. \end{aligned}$$

Let’s call $RR_{r}$ the reference *RR* interval associated to a reference heart beat and $RR_{i}$ the one to the *i*-th *RR* interval from one beat at the *i*-th segment, then

11 $$\begin{aligned} QT_{i}-QT_{r}= \beta \Big ( (RR_{i})^{\alpha } - (RR_{r})^{\alpha } \Big ). \end{aligned}$$

As the $QT_{i}-QT_{r}$ difference, also $d_{w} (i)$ is a measure of width change between the reference *r* and the current *i*-th mean T-waves from their respective observations time windows, then it is possible to extend previous relation in (11) to $d_{w}(i)$ obtaining the HR related component

12 $$\begin{aligned} d_{w,HR}(i)= \beta \Big ( (RR_{i})^{\alpha } - (RR_{r})^{\alpha } \Big ). \end{aligned}$$

By substituting (12) in (9) we obtain

13 $$\begin{aligned} d_{w}(i)= d_{w,c}(i)+\beta \Big ( (RR_{i})^{\alpha } - (RR_{r})^{\alpha } \Big ). \end{aligned}$$

The value $d_{w,c}(i)$ can be assumed to be non-zero mean, and uncorrelated to HR, that is:

14 $$\begin{aligned} d_{w,c}(i) = b + \Delta d_{w,c}(i), \end{aligned}$$

with $\Delta d_{w,c}(i)$ zero mean and uncorrelated to HR. Then, $d_{w}(i)$ becomes:

15 $$\begin{aligned} d_{w}(i)= b + \Delta d_{w,c}(i) +\beta \Big ( (RR_{i})^{\alpha } - (RR_{r})^{\alpha } \Big ), \end{aligned}$$

where the parameters *b*, $\beta$ and $\alpha$, once jointly estimated (i.e. ${\hat{b}}$, ${\hat{\beta }}$ and ${\hat{\alpha }}$) can be used to derive ${\hat{d}}_{w,c}(i)$ as:

16 $$\begin{aligned} {\hat{d}}_{w,c}(i)= d_{w}(i)- {\hat{\beta }} \Big ( (RR_{i})^{{\hat{\alpha }}} - (RR_{r})^{{\hat{\alpha }}} \Big ). \end{aligned}$$

Note that, ${\hat{\beta }}$ and ${\hat{\alpha }}$ cannot be assessed from (15) with a directly least square fitting, since the DC component *b* in (15) largely affects the results. Rather, it is possible to jointly estimate ${\hat{b}}, {\hat{\beta }}$ and ${\hat{\alpha }}$, and then use the results in (16).

This estimate can be further approximated linearly for small RR changes. Denoting $\Delta RR(i)=RR_{i} - RR_{r}$, $RR_{i}$ can be expressed as $RR_{i}= RR_{r}+ \Delta RR(i)$ and by replacing this in the right side of (12):

17 $$\begin{aligned} d_{w,HR}(i)= \beta \Big ( ( RR_{r}+ \Delta RR(i))^{\alpha } - (RR_{r})^{\alpha } \Big ). \end{aligned}$$

Operating on the terms and under the assumption that $\Delta RR(i) \ll RR_{r}$ , $(\frac{ \Delta RR(i)}{RR_{r}}) \ll 1$ and by using the Taylor’s series expansion, we have

18 $$\begin{aligned} (RR_{r}+ \Delta RR(i))^{\alpha } - (RR_{r})^{\alpha } \simeq \alpha \Delta RR(i) (RR_{r})^{(\alpha -1)}. \end{aligned}$$

Substituting (18) in (13):

19 $$\begin{aligned} d_{w}(i) \simeq b + \Delta d_{w,c}(i)+\alpha \ \beta \ \Delta RR(i) (RR_{r})^{(\alpha -1)}, \end{aligned}$$

where *b*, $\alpha$, $\beta$ and $(RR_{r})^{(\alpha -1)}$ are constant values; then placing:

20 $$\begin{aligned} \alpha \ \beta (RR_{r})^{(\alpha -1)} = c, \end{aligned}$$

the actual $d_{w}(i)$ dependency with RR will be:

21 $$\begin{aligned} d_{w}(i) \simeq b + \Delta d_{w,c}(i)+c \ \Delta RR(i). \end{aligned}$$

**Figure 3:**
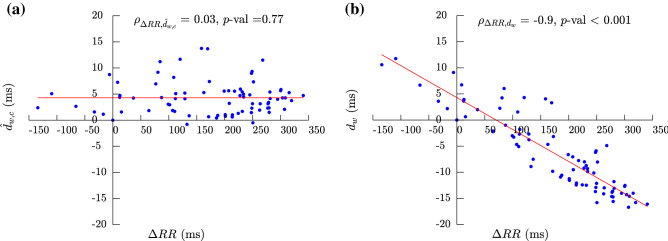
Scatterplot showing the values of both $d_w$ panel (**a**) and ${\hat{d}}_{w,c}$ panel (**b**) with respect to $\Delta RR$ for a given patient in PCA approach. Spearman’s correlation coefficients ($\rho$) and *p*-values for both $d_w$ and ${\hat{d}}_{w,c}$ are shown on top of each panel, while the least-square fitting regression lines are plotted in red.

From the geometrical point of view, *b* and *c* can be estimated as the zero-crossing and the slope, respectively, of the least-squares line fit to the $d_{w}(i)$ values (in a $\Delta RR(i)$ vs. $d_{w}(i)$ graph). Then, the $d_{w}(i)$ component that does not dependent on the RR, meaning it is assumed not correlated, can assessed as:

22 $$\begin{aligned} {\hat{d}}_{w,c}(i)= d_{w}(i)- {\hat{c}} \ \Delta RR(i) = d_{w}(i)-{\hat{c}}(RR_{i} - RR_{r}). \end{aligned}$$

where ${\hat{c}}$ is the estimated slope from the Holter recording, see Fig. 3, ${\hat{d}}_{w,c}(i)$ is then the corrected estimated of $d_{w,c}(i)$, with $RR_{i}$ and $RR_{r}$ the mean RR interval from the *i*-th studied segment and the reference windows respectively and ${\hat{c}}$ parameter is estimated for every patient during the time course of the Holter recording. When the linear approximation presented above cannot be assumed, ${\hat{b}}$, ${\hat{\beta }}$, and ${\hat{\alpha }}$ can be jointly estimated from the model in (15), and use the (16) as the corrected estimate.

An example of the estimated ${\hat{d}}_{w,c}(i)$ is given in Fig. 5a where both ${\hat{d}}_{w,c}(i)$ and $d_w(i)$ where displayed. Notice how the proposed correction formula removed the HR-dependency, for example around h = 12.

Table 3 provides an overview of the morphology markers studied in this work.

**Table 1:** Characteristics of the study population.

	(N = 29)
Age (years)	$75\ (12)$
Gender (male)	$20\ (70\%)$
Anti-arrhythmic drugs (yes)	$9\ (31\%)$
Implanted pace-maker (yes)	$1\ (3\%)$
Time under HD treatment (months)	$15\ (59)$
**HD session duration**	
210 min	$3\ (10\%)$
240 min	$26\ (90\%)$
**Kidney disease etiology**	
Diabetes mellitus	$17\ (59\%)$
Interstitial nephritis	$2\ (7\%)$
Glomerulonephritis	$2\ (7\%)$
Tuberous sclerosis	$1\ (3\%)$
Polycystic kidney	$1\ (3\%)$
Cancer	$1\ (3\%)$
Unknown	$5\ (18\%)$
**HD liquid composition**	
Potassium (1.5 mmol/L)	$21\ (72\%)$
Potassium (3 mmol/L)	$5\ (17\%)$
Potassium (decreasing)	$3\ (11\%)$
Calcium (2.5 mg/dL)	$21\ (72\%)$
Calcium (3 mg/dL)	$8\ (28\%)$
**HD techniques**	
Conventional	$18\ (62\%)$
Online	$8\ (28\%)$
Acetate-free biofiltration with decreasing intra-HD $[K^{+}]$	$3\ (10\%)$

Values are expressed as *number* ($\%$) for categorical variables, and *median* (IQR) for continuous variables.

**Table 2:** Blood potassium concentration $[K^{+}]$ values (in mmol/L) at each blood extraction during the HD ($h_0$ to $h_4$) and HR (beats/min).

	$h_0$	$h_1$	$h_2$	$h_3$	$h_4$	$\rho$	*r*
($K^{+}$)	5.0 (1.4)	3.8 (1.1)	3.6 (0.8)	3.4 (0.7)	3.3 (0.6)	0.10 (1.35)	0.09 (1.45)
HR	81 (28)	76 (28)	80 (23)	80 (17)	80 (25)		

Spearman’s ($\rho$) and Pearson’s (*r*) intra-patient correlation coefficients between $[K^{+}]$ and RR. Values are expressed as median (IQR).

**Table 3:** T-wave morphology markers for $[K^{+}]$ monitoring.

Markers	Description
**Original markers from**^17^	
$d_{w}^{u}$*	Time-domain changes between
	The reference and the *i*-th MWTW (ms).
$d_w^{\mathrm{NL}}$	Nonlinear component of the
	Time-domain changes between
	The reference and the *i*-th MWTW (ms)
$d_a$	Relative amplitude changes between
	The reference and the *i*-th MWTW (%)
$d_a^{\mathrm{NL}}$	Relative nonlinear amplitude changes
	After normalising the reference
	And the *i*-th MWTW (%)
**Specifically proposed in this work**	
$d_{w}$	Signed version of the
	Previously proposed $d_{w}^{u}$* (ms)
$d_{w,c}$	Heart rate corrected version of $d_{w}$ (ms)

*$d_{w}^{u}$ correspond to the marker denoted as $d_{w}$ in^17^, while here.

$d_{w}$ is reserved for the newly introduced signed version.

### Potassium concentration variations $\Delta [K^+]$

The proposed biomarkers have been compared with the relative variations in $[K^+]$ (denoted as $\Delta [K^+](h)$) with respect to a reference $[K^+]$ that was taken at the end of the HD:

23 $$\begin{aligned} \Delta [K^{+}](h) = ([K^{+}]_{h} - [K^{+}]_{r}) \end{aligned}$$

being $[K^{+}]_{h}$ the concentration at the *h*-th hour during the HD and $[K^{+}]_{r}$ the concentration at the end of the treatment. An example of the $\Delta [K^+](h)$ evolution is shown in Fig. 5a (purple diamonds).

### Statistical analyses

Results are presented as median and interquartile range (IQR). Spearman rank correlation coefficient ($\rho$) and Pearson correlation (*r*) were used for correlation analysis between $\Delta [K^+]$ and the proposed biomarker, giving information about both the monotonic relation and the strength of the association between the time warping based biomarkers and $[K^+]$ changes and then providing a more complete characterisation. The average duration of the ECG recordings was 44 h mainly due to electrode detachment or early battery exhaustion. For this reason, correlation coefficients were computed using the first five values of $\Delta [K^{+}](h)$ throughout the HD and the warping markers evaluated at the corresponding *i*-th segment points ($h=(i-1)/2$ where *i*=1, 3, 5, 7, 9 or *i*=1, 3, 5, 7, 8 depending on the HD duration). All statistical analyses were performed using MATLAB version R2018b.

## Results

In this study, ECG signals and $[K^+]$ from 29 ESRD-HD patients were investigated. An example of $d_{w}$ and ${\hat{d}}_{w,c}$ time evolution for a particular patient, in PCA approach, was provided in Fig. 3. $\Delta RR$ was represented on the x-axis in both panels, while $d_{w}$ and ${\hat{d}}_{w,c}$ were shown on the y-axis in panel (a) and panel (b), respectively. The least-square fitting line (red line) was depicted in both panels. Spearman’s correlation coefficients ($\rho$) and *p*-values were also showed in each panel. High and significant correlation ($\rho =-\,0.90$ and *p*-value $<0.001$) was found between $\Delta RR$ and $d_{w}$. However, after correcting for the HR-dependency, $\rho =0.03$ and *p*-value $=0.76$.

Correlation between $[K^+]$ and mean HR expressed in beats per minute (bpm) have also been computed and the results are presented in Table 2, with a Spearman’s correlation coefficient median (IQR) values of 0.10 (1.35), and a median *p*-value of *p*=0.33. These values were 0.09 (1.45), *p* = 0.22 for Pearson’s correlation coefficient.

Table 4 shows the intra-patient Spearman’s ($\rho$) and Pearson’s (*r*) correlation coefficients computed between the relative variations in $[K^+]$ (denoted as $\Delta [K^+]$) with respect to a reference $[K^+]$ that was taken at the end of the HD and the time-warping parameters. In both single-lead and PCA approaches, the highest median Spearman’s and Pearson’s correlation coefficients were found for $d_{w}^{u}$, $d_{w}$ and ${d}_{w,c}$ being $\rho \ge 0.82$ and $r \ge 0.86$ for single-lead analysis and $\rho \ge 0.82$ and $r \ge 0.89$ in PCA.

**Figure 4:**
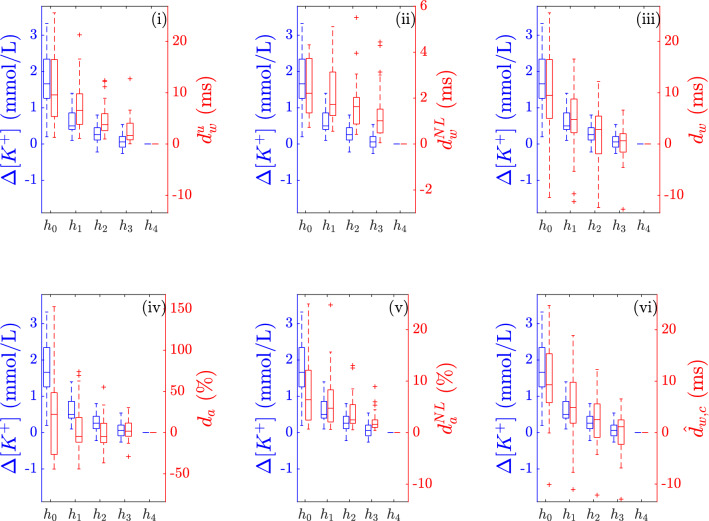
Boxplots showing the distribution of $\Delta [K^{+}]$ (blue) and all the described PCA-based time-warping biomarkers ($d_{w}^{u}$, $d_a$, $d_w^{\mathrm{NL}}$, $d_a^{\mathrm{NL}}$, $d_{w}$, and ${\hat{d}}_{w,c}$) (red), computed at different time points from the beginning, $h_0$, to the end, $h_4$, of the HD session ($h_0$ to $h_4$ in Fig. 1). $\Delta [K^{+}]$ was computed as in (23). + denotes outliers.

**Figure 5:**
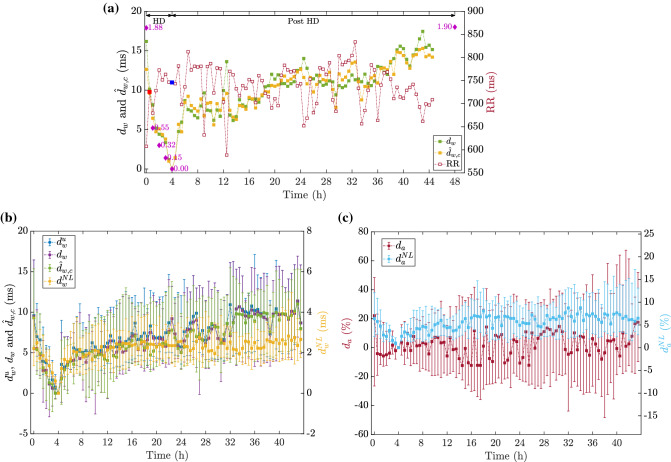
PCA-based time-warping markers and RR interval time trends. An example for a given patient undergoing 4h-long HD therapy, is depicted in panel (**a**) with the evolution of $d_{w}$ (filled green squares), ${\hat{d}}_{w,c}$ (filled orange squares), both referring to the left vertical scale, and the average RR intervals (unfilled dark red squares, referring to the right vertical scale). $\Delta [K^{+}]$ relative variations with respect to the concentration at HD end (purple diamonds) are expressed in mmol/L. Time is expressed in hours from the beginning of the treatment onward. Each square denotes the mean RR interval in a 2-min wide segment used to compute the warping parameters, while the highlighted blue square corresponds to the reference segment at the end of HD. The filled red square denotes the time-point from which the studied MWTW in Fig. 2b was selected. Note that for this patient, the Holter recording did not reach the planned 48h. Panel (**b**) shows the median and IQR for each observing *i*-th segment, computed by using the values from all the available patients for $d_{w}^{u}$, $d_w$ and ${\hat{d}}_{w,c}$ and $d_{w}^{\mathrm{NL}}$ (this latter refers to the right axis, while the others to the left). Time trends (expressed as median and IQR) for $d_{a}$ and $d_{a}^{\mathrm{NL}}$, referring to the left and right axes respectively are in panel (**c**). Panels (**b**) and (**c**) give an overview of the time evolution for these biomarkers along the ECG acquisition from the HD beginning onward. Only the first 44 h were depicted being that the average ECG duration in our database.

Boxplots in Fig. 4 show the distributions of $\Delta [K^{+}]$ and the proposed PCA-based time-warping descriptors during HD. Figure 5b shows the average time evolution of PCA-based $d_{w}^{u}$, $d_w$, ${\hat{d}}_{w,c}$ and $d_{w}^{\mathrm{NL}}$ in the studied population along the monitoring period, while the evolution of $d_a$ and $d_{a}^{\mathrm{NL}}$ is shown Fig. 5c.

**Table 4:** Intra-patient Spearman’s ($\rho$) and Pearson’s (*r*) correlation coefficients between $\Delta [K^{+}]$, and time-warping based markers, and $T_{w}$ and $T_{S/A}$, in all cases evaluated from the first PCA transformed lead, and from standard single leads II, V3, V4, V5 and V6. Values are expressed as median (IQR).

	Spearman’s ($\rho$)						Pearson’s (*r*)					
	PCA	II	V3	V4	V5	V6	PCA	II	V3	V4	V5	V6
$d_{w}^{u}$	0.90 (0.37)	0.90 (0.40)	0.86 (0.50)	0.90 (0.38)	0.90 (0.25)	0.90 (0.29)	0.92 (0.36)	0.91 (0.37)	0.85 (0.37)	0.86 (0.41)	0.92 (0.14)	0.86 (0.30)
$d_{w}$	0.82 (0.45)	0.90 (0.37)	0.90 (0.49)	0.90 (0.46)	0.90 (0.30)	0.90 (0.39)	0.89 (0.35)	0.90 (0.26)	0.88 (0.31)	0.86 (0.39)	0.93 (0.11)	0.87 (0.29)
${\hat{d}}_{w,c}$	0.90 (0.31)	0.82 (0.28)	0.86 (0.40)	0.90 (0.50)	0.90 (0.30)	0.90 (0.38)	0.89 (0.25)	0.87 (0.33)	0.89 (0.29)	0.86 (0.49)	0.91 (0.17)	0.86 (0.27)
$d_{w}^{\mathrm{NL}}$	0.82 (0.57)	0.43 (0.60)	0.70 (0.45)	0.67 (0.60)	0.70 (0.64)	0.80 (0.56)	0.68 (0.52)	0.47 (0.58)	0.61 (0.47)	0.56 (0.45)	0.56 (0.64)	0.61 (0.47)
$d_a$	0.21 (1.20)	0.00 (1.22)	0.50 (0.80)	0.40 (1.45)	0.45 (1.59)	− 0.10 (1.15)	0.57 (1.53)	0.14 (1.55)	0.83 (0.67)	0.63 (1.71)	0.62 (1.65)	− 0.32 (1.52)
$d_{a}^{\mathrm{NL}}$	0.80 (0.42)	0.65 (0.70)	0.70 (0.59)	0.70 (0.77)	0.86 (0.45)	0.87 (0.40)	0.75 (0.39)	0.82 (0.83)	0.66 (0.56)	0.68 (0.84)	0.88 (0.30)	0.84 (0.38)
$T_{w}$	− 0.70 (0.59)	− 0.84 (0.57)	− 0.90 (0.52)	− 0.75 (0.67)	− 0.80 (0.63)	− 0.90 (0.65)	− 0.92 (0.32)	− 0.85 (0.35)	− 0.87 (0.40)	− 0.81 (0.62)	− 0.84 (0.44)	− 0.75 (0.48)
$T_{S/A}$	0.80 (0.42)	0.62 (0.80)	0.60 (0.85)	0.46 (1.07)	0.76 (0.59)	0.63 (0.69)	0.86 (0.38)	0.83 (0.77)	0.71 (0.65)	0.46 (0.83)	0.74 (0.45)	0.72 (0.52)

## Discussion

Repolarization abnormalities play a fundamental role in the genesis of arrhythmic events and the risk increases in patients at ESRD with imbalance in $[K^{+}]$^41^. In this work, two previously reported potassium estimators, $T_{w}$^14^ and $T_{S/A}$^15^, four warping-based ECG-derived biomarkers for $[K^{+}]$ monitoring proposed in Ramírez et al.^17^, $d_{w}^{u}$, $d_a$, $d_w^{\mathrm{NL}}$, $d_a^{\mathrm{NL}}$, and the here proposed modified versions $d_{w}$ and ${d}_{w,c}$ were tested as bloodless indices for $[K^{+}]$ variations in ESRD-HD patients computed from standard leads as well as in a PCA-derived lead. The most promising results in terms of correlation were obtained for markers $d_{w}^{u}$, $d_{w}$, and ${d}_{w,c}$, leading to the highest median intra-patient $\rho \ge 0.82$ and $r \ge 0.87$ in single-lead and $\rho \ge 0.82$ and $r \ge 0.89$ in PCA lead respectively, evidencing high monotonic and linear association with $[K^{+}]$ and making them a promising non-invasive indices for blood $[K^{+}]$ monitoring.

The signed biomarker $d_{w}$ followed a similar time-course as the unsigned $d_{w}^{u}$ during the whole monitoring period, showing a similar distribution in Fig. 5a, as a result of the fact that the sign computed as in (5) is positive in roughly all the patients. That can be explained by the fact that the T-wave morphology in hyperkalemia is usually more peaked and shorter in time than a T-wave from regular $[K^{+}]$ concentrations, as happens at the end of HD, where the reference has been taken^42,43^. Therefore, all the other MWTWs needed to be shrunk in amplitude and widened in time duration during the warping procedure to fit the reference one, and this is given by a positive signed $d_{w}$. However, other external factors, such as the potassium removal rates^44^ or the dialysate potassium level^45,46^, might also have played a role in altering ventricular repolarization activity.

The warping algorithm is applied over the MWTWs computed from different observing windows with different HRs, as is evident in Fig. 5a. Therefore, a corrected version of the $d_{w}$, derived similarly to the QT correction formula^38,39^, was proposed since the HR influences this marker as pointed out in Ramírez et al.^17^, and can be observed in Fig. 5a as an example around hours h$=$9, 12 and 43. A large number of models have been proposed for the computation of QTc values independent of HR^37–40^. However, a previous study^38^ found that the linear regression model fits better than any other model to the relationship between QT and the RR intervals. Also, for small RR variations, in section “Heart-rate-corrected T-wave warping” it is shown that hyperbolic QT to RR dependency becomes linear. Therefore, we used a linear model to derive an HR-corrected index, ${d}_{w,c}$. This approach was used to estimate the $d_{w}$ component strictly related to $[K^{+}]$ removing its relation with HR as showed in Fig. 3, where the HR-dependency, clearly visible in panel (a), was cancelled after the correction, panel (b). Comparing the results for $d_{w}^{u}$, $d_{w}$ and ${\hat{d}}_{w,c}$, all of them have proved to be highly correlated with $[K^{+}]$ variations. However, it is important to remember that $d_{w}^{u}$ (and so $d_{w}$) is biased by the HR effects as previously described^17^, while ${\hat{d}}_{w,c}$ is no longer dependent on it, possibly being responsible for the lower IQR in the correlation, 0.25, as compared to 0.35 and 0.36 for $d_{w}$ and $d_{w}^{u}$, respectively (see Table 4, PCA column). It should also be noted that the small differences between the $\rho$ and *r* computed for ${\hat{d}}_{w,c}$ and $d_w$ could be due to the low HR variations observed during HD, but larger HR variations, and consequently a higher impact of the correction, are expected in ambulatory monitoring.

In addition, we investigated the correlation between HR and $[K^{+}]$ finding no significant correlation between the two. To place these results into a proper context, in a previous study on computer-based models it was found that the heart rate in ESRD-HD patients is influenced by the combined effects of $[K^{+}]$), calcium and pH^47^. In particular, it was observed that when $[K^{+}]$ is between 3 and 4 mmol/L, the HR sensitivity is about 10 bpm/mmol of $[K^{+}]$^47^. As can be seen in Table 2, the median $[K^{+}]$ falls within the above mentioned range from $h_1$ to $h_4$ (i.e., almost during the entire HD session) but the range of HR variations is much bigger than the 10 bpm mentioned in Severi et al.^47^ (minimum IQR = 17 bpm at $h_3$) so anticipating the obtained low and insignificant median correlations coefficients between HR and $[K^+]$. This result suggests that HR variations are a poor indicator of $\Delta [K^+]$, at least in our dataset. Moreover, this finding gives validity to the proposal of the ${\hat{d}}_{w,c}$ presented in this work, which assumes no correlation between HR and $[K^{+}]$-related changes on T-wave morphology.

In a previous study^20^ a comparison of $T_w$, $T_{S/A}$, $d_{w}$, $d_w^{\mathrm{NL}}$, $d_a$ and $d_a^{\mathrm{NL}}$, based on a electrophysiological simulation of ECG under hyperkalemia, was performed, and tested on a subset of the ESRD-HD patients described in Materials and Methods section. In that study it is shown that similar results were obtained in terms of Pearson’s correlation coefficient between $[K^{+}]$ and $T_w$, $T_{S/A}$, and time-warping based markers, also showing a high correlation with HR. In the present work we extended the analysis by increasing the sample size, introducing the proposed correction by HR, and comparing with single lead recordings. According to the Spearman’s ($\rho$) and Pearson’s (*r*) correlation coefficients, the median values are higher, and the IQR smaller, for $d_{w}^{u}$, $d_{w}$ and ${d}_{w,c}$ than for $T_{S/A}$ using both PCA and standard single-lead approaches. For $T_w$, we found similar *r* median absolute values, as compared to $d_{w}^{u}$,$d_{w}$ and ${d}_{w,c}$, while Spearman’s correlation values were much lower for $T_w$ when using PCA and several single-leads (e.g. lead V4 and V5). This shows that the proposed time-warping based markers present either higher correlation or stronger monotonic relationship with $\Delta [K^+]$ than $T_w$, and $T_{S/A}$, making them more suitable for $[K^{+}]$ monitoring purposes.

When comparing single-lead and PCA-based analysis, and according to Spearman’s ($\rho$) and Pearson’s (*r*) correlation coefficients in Table 4, leads II, V3 and V5 are those showing the highest median and the smallest IQR values in a single-lead approach. Moreover, for $d_a$ and $d_a^{\mathrm{NL}}$, leads V3 and V5 show stronger $\rho$ and *r* correlation with $\Delta [K^+]$ than the PCA lead, with $\rho \ge 0.45$ and $r \ge 0.62$. However, median values for $\rho$ and *r* computed between $\Delta [K^+]$ and $d_{w}^{u}$, $d_{w}$ and ${d}_{w,c}$, are similar ($\rho \ge 0.82$ and $r \ge 0.89$ for the PCA lead and $\rho \ge 0.82$ and $r \ge 0.86$ for the standard single leads) showing no evident benefit in using one or the other strategy. Nevertheless, a clear advantage of extracting $d_w^{\mathrm{NL}}$ from the PCA lead rather than from the standard single leads can be observed when comparing the medians both from $\rho$ and *r*. In the same line, both $T_w$ and $T_{S/A}$ showed higher *r* computed from the PCA lead than from standard leads. This improvement when analysing biomarkers estimated from specific local features of the T-wave, as $T_w$ and $T_{S/A}$, which is not that evident when analyzing global T-wave waveform based biomarkers, like those proposed in this work, can be explained by the improved SNR obtained by using PCA, leading to a more robust definition of the T-wave delineation marks. This is in agreement with several studies reporting the benefits of PCA as an intermediate step when addressing problems related with noise reduction^28^. Therefore, the use of PCA from multilead ECG is recommended as a more robust alternative to single-lead analysis.

As already described, ECGs recorded under hyperkalemic conditions commonly present T-waves which are taller than those for normal levels of blood $[K^{+}]$. The marker $d_a$ was designed to capture the amplitude variability between ${\mathbf {f}}^i({\mathbf {t}}^i)$ and ${\mathbf {f}}^r({\mathbf {t}}^r)$ after warping. However, from its original definition, $d_a$ can take both positive and negative values, producing positive intra-patient $\rho$ and *r* between $\Delta [K^+]$ and $d_a$ in $62\%$ of the cases and negative in $38\%$ of the cases. This explains the low median inter-patient correlation ($\rho =0.21$) and the wide IQR (1.20). However, if its absolute value was considered then correlation between $\Delta [K^+]$ and $d_a$ becomes larger being $\rho =0.70$ (0.60) and $r=0.75$ (0.31).

Cellular AP duration can be affected in a heterogeneous manner by $[K^{+}]$ fluctuations since ion channel expression is heterogeneous throughout the ventricles^48^ and this can result in non-linear changes on T-wave morphology. According to their definition^17^, $d_{w}^{\mathrm{NL}}$ and $d_{a}^{\mathrm{NL}}$ were designed to quantify the inhomogeneous morphological variations during ventricular repolarization; this fact might explain their correlation with $\Delta [K^+]$. Both of them showed a remarkable sensitivity to the variations of ventricular spatio-temporal dispersion independently from changes in HR^17^, meaning that they did not need a HR correction as was done for $d_{w}$. This last point, particularly for $d_a^{\mathrm{NL}}$, in addition to its also high correlation with $\Delta [K^+]$, make it an interesting T-wave descriptor for blood $[K^{+}]$ assessment.

Results of the present work, show high accuracy of the $d_{w}$ ${d}_{w,c}$ T-wave based time-warping biomarkers for $[K^{+}]$ monitoring, in contrast with the findings in a recent study published by Regolisti et al.^49^ where lower correlation values were reported for other ECG-derived markers. The explanation for this discrepancy could be tracked back to the biomarkers the authors took as reference for their research, from Dillon et al.^13^ and Corsi et al.^15^, which are a) focused on specific features of the T-waves and b) measured in absolute value and not in relative terms to a reference which can personalize the biomarker as here presented. Nevertheless, time warping analysis was applied in recent studies^19, 20^ to investigate both hypo- and hyperkalemia on a extremely heterogeneous pool of simulated cases, performing equally well in all the cases and proving its adaptability, at least in silico simulated ECGs, probably due to its personalize profile as being comparative with a reference. Therefore, we hypothesized that the proposed methodology for $[K^{+}]$ monitoring could be applied to investigate a wide range of populations and experimental sets.

Other recent studies developed deep-learning models and tested them on large datasets to screen for hyperkalemia in patients with CKD, reporting a more robust recognition of severe hyperkalemia, thus potentially reducing the risk of sudden cardiac death in those patients^50,51^. However, in our study we aim, not just to detect hyperkalemia, but also in continuous $[K^+]$ quantification. Extension of the database will open the door to use deep learning techniques, which will allow proper comparison with the methodology here proposed.

The main limitation of this work concerns the reduced amount of blood tests (only six) took from each patient during the 48-h ECG recording, which provided an accurate representation of the $[K^{+}]$ time evolution only during HD, but not during the ambulatory period. On this basis, it was possible to investigate the relationship between the proposed markers and the $\Delta [K^+]$ only during the therapy but not through the remaining hours. Moreover, the course of HD procedures can be accompanied by ischaemia^52–54^, which can also be associated with changes in the T-wave, independently from HR, therefore reflected on $d_{w,c}$. Elucidation of its impact in monitoring normal-life outside the HD process needs to be explored in a dedicated study. Also postural or body position changes (BPC) are known to affect the T-wave morphology and mainly the T-wave amplitude^55^, which can influence $d_a$ related biomarkers. The fact that those markers were measured on PCA lead could attenuate the effect of BPC on T-wave, however a specific study should precisely establish the impact of this aspect on the biomarker, particularly when applied in a non controlled scenario. Assessment of the performance of the proposed descriptors in patients with hypokalemia remains to be studied. In addition, the methodology proposed in this work needs to be validated in: (a) a larger population; and (b) subsequent HD sessions on the same population, which would allow to quantify the accuracy in $[K^{+}]$ estimation.

Beyond the previous considerations, $d_{w}$, ${\hat{d}}_{w,c}$ and $d_{a}^{\mathrm{NL}}$ showed potential value in monitoring changes in blood potassium concentration. All of them showed a time-course similar to that of $[K^{+}]$ in ESRD-HD patients as described in literature^45,46^: a rapid decline during the HD with a fast rebound just after the end of the therapy, followed by a steady increase in the remaining hours before the next HD session (see Fig. 5b,c). Therefore, it is possible to consider the biomarkers use for continuous $[K^{+}]$ monitoring, also in other pathologies as in patients suffering heart failure^56^, where hyperkalemia also increases the risk for SCD.

As a future study, we propose to replicate the same analysis over a larger population and assess the correlation between the proposed time-warping markers and alterations in other electrolytes beyond $[K^{+}]$, like calcium, magnesium, or related to the rate and amount of $[K^{+}]$ removal^44–46^. These variations could alter the ECG and it would be interesting to analyse their relationship with the induced T-wave changes. Then, as mentioned above, there is a need to test the proposed indices and investigate their robustness against these possible confounders. Other alternatives for lead space reduction, such as Periodic Component Analysis^57^, can be explored to check whether the T-wave shape is better emphasized with a criterion different from just the energy maximisation considered by PCA. A study to test the extrapolation power of the current findings, which refer to $[K^+]$ levels at $h_4$, to subsequent HD sessions should also be performed to assess their clinical long-term validity.

## Conclusions

In this study, time-warping markers ($d_{w}$, $d_a$), their non linear components ($d_{w}^{\mathrm{NL}}$ and $d_{a}^{\mathrm{NL}}$) and a HR-corrected version $d_{w,c}$, in all cases personalised making it relative to reference point, were studied in ESRD patients undergoing HD and evaluated as estimator of $[K^{+}]$ changes over time. Among the analyzed biomarkers and methods (i.e. standard single-lead or PCA transformed lead approaches), the proposed PCA-based $d_{w}$ and its HR corrected version, ${d}_{w,c}$, achieved better results than the previously proposed $T_{S/A}$ in terms of both Spearman’s and Pearson’s correlation with $\Delta [K^+]$, and showed higher monotonic relationship than $T_w$, thus making the proposed time-warping markers a valid and more accurate alternative to the currently available tools for $[K^{+}]$ ambulatory monitoring of ESRD-HD patients. In conclusion, this study proposes new markers for robust quantitative ambulatory monitoring of $[K^{+}]$ from the ECG. The proposed markers could improve routine $[K^{+}]$ monitoring without the need of invasive blood test, both in hospital and in an ambulatory scenario. A comprehensive validation is needed to corroborate the extrapolation power of these results.
